# Methane oxidation in lead-contaminated mineral soils under different moisture levels

**DOI:** 10.1007/s11356-017-0195-8

**Published:** 2017-09-20

**Authors:** Ewa Wnuk, Anna Walkiewicz, Andrzej Bieganowski

**Affiliations:** Department of Natural Environment Biogeochemistry, Institute of Agrophysics, Polish Academy of Sciences, Doświadczalna 4, 20-290 Lublin, Poland

**Keywords:** Methane, Heavy metals, Soil moisture, Lead, Methane oxidation, Methanotrophic activity

## Abstract

Methane (CH_4_) oxidation in soil reduces the concentration of this greenhouse gas due to the activity of methanotrophic bacteria. This process is influenced by chemical and physical parameters of soil. We tested the methanotrophic activity of selected mineral soils (Mollic Gleysol, Haplic Podzol, Eutric Cambisol) contaminated with lead (Pb) under different soil water potentials (pF 0; 2.2; 3.2). The heavy metal was added as PbCl_2_ in two doses. Together with the initial content of Pb in soils, the final contents of heavy metal in different soils were 11.6 and 30.8 mg kg^−1^ in Eutric Cambisol, 7.1 and 26.3 mg kg^−1^ in Haplic Podzol, and 12.2 and 31.4 mg kg^−1^ in Mollic Gleysol (dry mass of the soil is specified in all cases). The results showed relatively low sensitivity of methane oxidation to the addition of the heavy metal. The major factor controlling this process was soil water content, which in most cases turned out to be the most optimal at pF = 2.2.

## Introduction

The problem of greenhouse gas (GHG) emission in the context of climate changes is still very urgent (Serrano-Silva et al. 2014). Different sources of GHGs are being investigated currently (Smith and Conen 2004; Pytlak et al. 2014). A big effort made by researchers is focused on the issue of GHG emission (Nosalewicz et al. 2013; Haas et al. 2016; Jain et al. 2016). The literature review leads to a conclusion that a relatively small number of investigations concerning all GHGs are devoted to methane in the context of contaminated soils.

Methane (CH_4_) is one of the most important trace gases with a global warming potential (GWP_100_) 28 times greater than that of carbon dioxide (CO_2_) (IPCC 2014). For the last 200 years, the concentration of this gas has doubled, due to the increase in its emission caused by anthropogenic sources such as ruminant husbandry, rice production, fossil fuel excavation, and burning or utilization of wastes. Another reason is the climate change-driven imbalance between CH_4_ emission and oxidation (Nosalewicz et al. 2011; Contin et al. 2012; Frąc and Ziemiński 2012; Walkiewicz et al. 2016).

Soil is a biosphere element that plays an important role in methane circulation. This ecosystem fulfills two contradictory functions (CH_4_ production and oxidation) depending on the oxygen conditions. Under aerobic conditions, the process of methane oxidation is carried out by methanotrophs (methane-oxidizing bacteria) which naturally colonize the soil. They use CH_4_ as a source of carbon and energy to carry out further reactions (Grosso Del et al. 2000; Einola et al. 2007). Provided with favorable conditions, methanotrophs are extremely effective in methane turnover. They are also known to be able to survive long-term starvation, desiccation, and oxygen depletion (Stępniewska et al. 2013, 2014).

Associated with oxygenation, soil water content is one of the most important elements, together with temperature, which controls many processes in soil (Sławiński et al. 2012; Kaczmarek et al. 2016; Lukowska and Józefaciuk 2016). By changing aeration, soil moisture determines two opposite processes. Total flooding results in anaerobic conditions, which activate methanogens producing CH_4_ (Das and Adhya 2012). Lower water content and partial filling of pores with air creates conditions suitable for aerobic methanotrophs. Moisture affects their activity by regulating gas diffusion—including O_2_ and CH_4_ (Boeckx and Van Cleemput 2000; Neira et al. 2015). The optimum moisture for methanotrophic activity depends on the content of soil organic matter as well as the ecosystem and land-use.

Heavy metals are one of the most important pollutants, which can remain in soil for many years. The most popular heavy metal pollutants are Cu, Ni, Pb, Cd, Cr, and Zn (Brookes 1995; Zgłobicki et al. 2015). Heavy metals enter the soil from many sources e.g., transport, mining, smelting, sewage sludge, or industrial and agricultural practices (Brookes 1995; Mohanty et al. 2000; Pawłowska et al. 2011; Medyńska-Juraszek and Kabała 2012).

It is obvious that the abovementioned factors i.e., water content (and resulting oxygen conditions) and heavy metals influence the methanotrophic efficiency of the soil. There are few papers describing the influence of different stress conditions on methanotrophic activity (Durisch-Kaiser et al. 2005; Wang et al. 2011). However, the issue has not been fully explained and described; moreover, there are no investigations focused on the combination of stressors.

The aim of this study was to determine the effect of stressors, i.e., different moisture levels (and the resulting differentiated oxygen concentration) and lead contamination, on methane oxidation in three mineral soils.

## Materials and methods

### Soil characteristics

Three mineral soils typical for South East Europe—Mollic Gleysol, Haplic Podzol, and Eutric Cambisol—were collected from a depth of 0–20 cm, air-dried, and sieved to < 2 mm. To obtain the representative soil sample (averaged sample), over a dozen point sub-samplings were carefully mixed. The basic soil properties are presented in Table 1. The criterion for soil selection was similar texture to establish similar in situ soil air-water conditions.

**Table 1:** Basic properties of tested soils (Walkiewicz et al. 2012)

Soil types		Eutric Cambisol	Haplic Podzol	Mollic Gleysol
Place of sampling (Poland)		Bonin (Zachodniopomorskie voivodeship)	Olsza (Kujawsko-Pomorskie voivodeship)	Sobocka Wieś (Łódzkie voivodeship)
Particle size distribution (%, dia in μm]	2000–50	71.6	74.6	74.8
	50–2	25.1	22.3	21.7
	< 2	3.23	3.04	3.44
C_org_ (%)		1.18	0.43	3.93
pH (KCl)		6.38	6.5	7.71
Pb (mg kg^−1^)		6.87	2.32	7.49
N (%)		0.08	0.09	0.17
NH_4_^+^ (mg kg^−1^)		4.20	0.49	2.84
P (mg kg^−1^)		180	30	297
K (mg kg^−1^)		93	200	127
K_m_ (μmol)		5.98	19.79	30.66
V_max_ (μmol g^−1^ h^−1^)		0.137	0.443	0.550

Organic carbon (C_org_) was determined using a TOC-VCPH analyzer (Shimadzu, Japan). Soil pH was measured potentiometrically in 1 mol KCl (1:2.5 *v*/*v*) after a 24-h stabilization at room temperature. Particle size distribution (PSD) was determined with the laser diffraction method with the use of Mastersizer 2000 (Malvern, UK) with a Hydro G dispersion unit (Polakowski et al. 2014). Pb contents in the soil samples were determined by ICP-OES (Inductively Coupled Plasma Optical Emission Spectrometry) from Thermo Scientific iCAP Series 6500, with a charge injection device (CID) detector (Kitowski et al. 2014).

### Incubation procedure

The experiment was based on the determination of CH_4_ consumption in lead-contaminated soil samples during incubation at three soil moisture levels corresponding to the values of soil water potential: pF = 0, pF = 2.2, and pF = 3.2 of each soil (pF is the measure of water holding capacity in soil pores). The amount of the added water solution corresponding to each pF was as follows: 30.02, 13.00, and 9.21% *v*/*v* in Eutric Cambisol; 35.14, 13.00, and 7.63% *v*/*v* in Mollic Gleysol; and 35.33, 13.00, and 4.52% *v*/*v* in Haplic Podzol, respectively. Lead (in the form of PbCl_2_) was added in the amounts corresponding to the limit values established by the Official Journal of the European Union in The Sewage Sludge Directive (86/278/EEC). Two doses were prepared—the maximum permitted dose of the metal (denoted as Pbx1–4.8 mg kg^−1^) and its fivefold higher dose (denoted as Pbx5–24 mg kg^−1^). Soil with addition of the CaCl_2_ solution was used as a control. The CaCl_2_ was added to soil in a sufficient amount to provide the same concentration of chloride ions corresponding to the concentration obtained in the case of lead salt to exclude the possible influence of chloride ions on the process of methane oxidation. The controls were analogous to the contaminated samples designated as CaCl_2_x1 and CaCl_2_x5.

Ten-gram samples of air-dried soil were weighed into 120 cm^3^ glass vessels and moistened with CaCl_2_ and PbCl_2_ solutions to moisture corresponding to the respective pF values. All the vessels were tightly closed with rubber stoppers and aluminum caps. Next, each vessel was enriched with 1% CH_4_ *v*/*v* in the headspace. The samples were incubated in the dark at 25 °C for 21 days. This temperature is optimum for CH_4_ oxidation (Xu and Inubushi 2009). Three independent replicates for each treatment were used.

### Gas concentration measurements and soil analysis

Consumption of CH_4_ and O_2_ and CO_2_ production in the headspace were measured with a gas chromatograph (Shimadzu GC-14A) with a thermal conductivity detector (TCD) using two columns (3.2 mm diameter): one packed with Porapak Q (for CH_4_ determination) and the other packed with Molecular Sieve 5A (for O_2_). Helium at a rate of 40 cm^3^ min^−1^ was used as a carrier gas. The temperature of the column and detector was 40 and 60 °C, respectively (Walkiewicz et al. 2012). Headspace gas (200 μl) of the same glass vessels was sampled for 21 days of the incubation.

Gas concentrations in the headspace were calculated based on the average of the triplicates. The average methane oxidation rate was calculated by subtracting the final concentration of the gas from the initial concentration and dividing the result by the number of incubation days.

All results were statistically analyzed using Statistica 10 software. The non-parametric Kruskal–Wallis test was used to determine the significance of the differences in the methane oxidation rate between the controls and contaminated samples at different soil moisture levels and heavy metal doses.

## Results

**Fig. 1:**
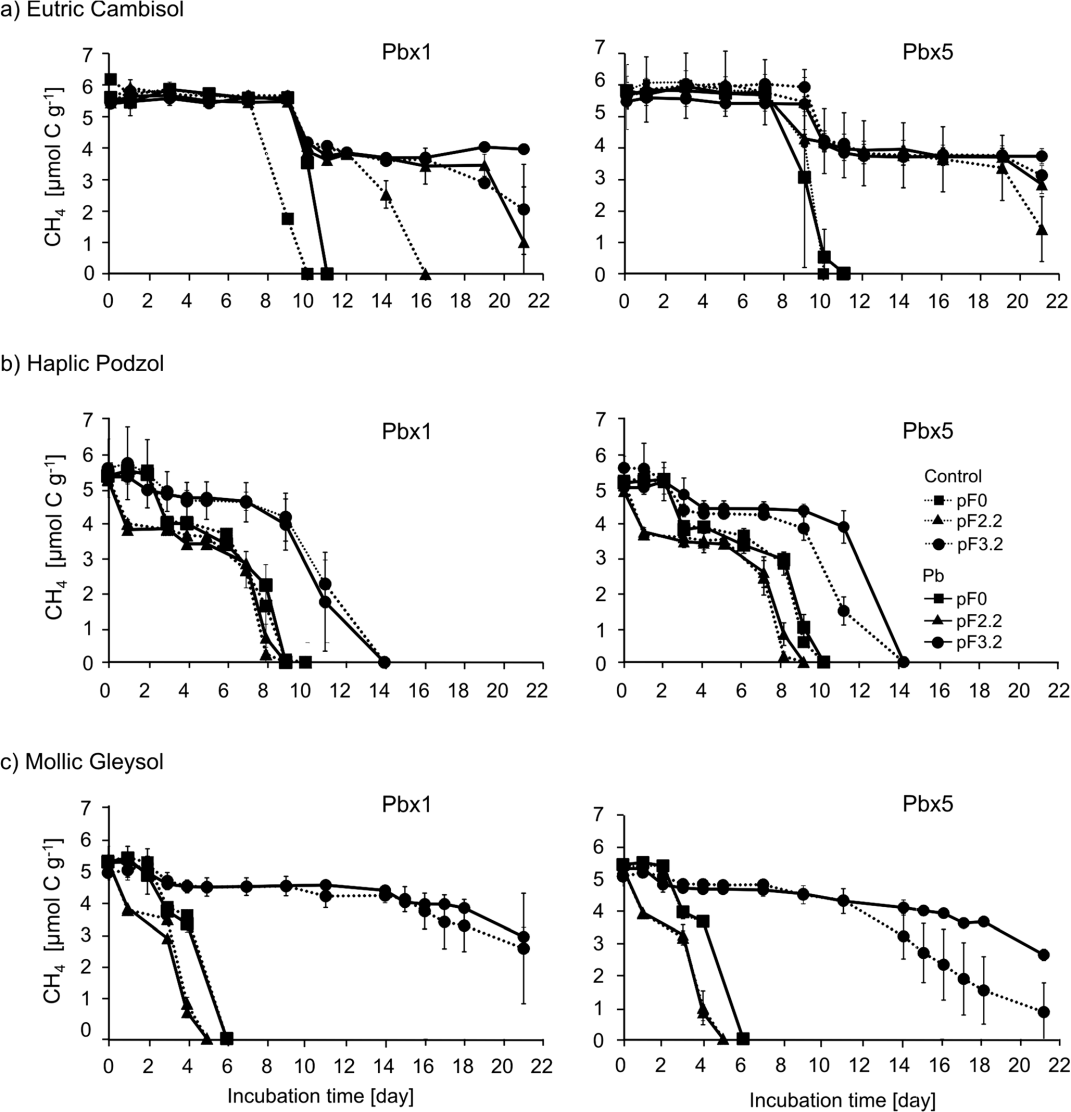
Decrease in the CH_4_ concentration with time in the headspace of the tested soils **a** Eutric Cambisol, **b** Haplic Podzol, and **c** Mollic Gleysol contaminated with Pb and in the control at three moisture levels pF = 0, pF = 2.2, and pF = 3.2. Heavy metal was added in two doses: the maximum permitted dose of the metal (Pbx 1) and the fivefold higher dose (Pbx 5). Points are averages of triplicate samples; bars indicate the standard deviations

The changes in the methane concentration in the headspaces of the incubated samples are shown in Fig. 1.

In general, in the controls, the least effective methane oxidation was carried out at pF 3.2, which is the lowest tested soil moisture. In Eutric Cambisol, the most effective methane oxidation was noted at pF 0. Complete oxidation of methane in the samples was carried out over 10 days in both Pb variants. In the third soil moisture (pF 2.2), all methane was oxidized only in a single control.

Haplic Podzol is the only soil in which all methane was consumed at all tested moisture levels. The fastest consumption, lasting 9 days, was recorded at pF 2.2, next at pF 0 over 10 days, and pF 3.2 over 14 days in both doses. Taking into account, the methane oxidation in Mollic Gleysol, the most favorable moisture for the process was observed at pF 2.2, where all methane consumption took 5 days. At pF 0, the time required to consume all methane was 6 days in both variants. At pF 3.2, methane was not oxidized completely during the 21 days of incubation. Comparison of both controls shows that the amount of oxidized methane is much higher at the fivefold higher dose than in the single control.

Comparison of the methane oxidation process in the contaminated soil and in the control revealed that methane consumption was only slightly changed by Pb addition. The most noticeable differences were observed in Eutric Cambisol. At pF 0, all methane was consumed over 11 days in both doses of the heavy metal, which is 1 day later in relation to the control. At pF 2.2, Eutric Cambisol contaminated with a single dose of Pb consumed 81.5% CH_4_ *v*/*v*, but it was completely oxidized in the corresponding control. At the lowest moisture content (pF 3.2), addition of a single dose of Pb resulted in oxidation of 25.9% of methane, which was ca. half the CH_4_ amount in comparison to the control. A similar tendency was observed in the same moisture conditions but with the higher Pb content (Pbx5), where 23.7% of CH_4_ was oxidized, which also accounted for ca. half the amount oxidized in the control. Addition of the fivefold dose of Pb to Eutric Cambisol at pF 2.2 resulted in 50% oxidation of the initial CH_4_ concentration, which was by ca. 25% lower than in the non-contaminated soil samples. All presented concentrations of oxidized CH_4_ refer to the tested incubation time (21 days).

In Haplic Podzol and Mollic Gleysol, methane was completely oxidized on the same day of incubation in both the control and contaminated samples. After Pbx5 addition to Haplic Podzol at pF 3.2, a quick decrease in the methane concentration occurred 1 day later than in the non-contaminated soil samples. Addition of Pb to Mollic Gleysol affected the final methane concentration at the lowest tested moisture (pF 3.2); in the Pbx1 variant, the gas was oxidized at a similar level (42.1–45.6% CH_4_). However, in Pbx5, the decrease in the final methane concentration was ca. 50% of CH_4_, which was ca. 30% lower than in the control.

The time required to start the process of methane oxidation (lag phase) was different in each moisture and type of soil. In Eutric Cambisol, the shortest lag phase lasting for 7 days was observed at pF 0; at the other moisture levels (pF 2.2 and 3.2), the lag phase ended after 9 days. After short 1-day gas consumption, the process was slowed down and completely finished at pF 2.2 for the single control. In Haplic Podzol, the shortest lag phase was observed at pF 2.2, and methane consumption started after 1-day adaptation to the prevailing conditions. At pF 0 and 3.2, the time required to start the process of methanotrophy was 3 and 2 days, respectively, in both controls. Analysis of the course of methane oxidation over time in Mollic Gleysol demonstrated that the process of methane oxidation at pF 2.2 started on the first day of incubation. At the other tested soil moisture levels, methane oxidation started after a 2-day lag phase.

**Fig. 2:**
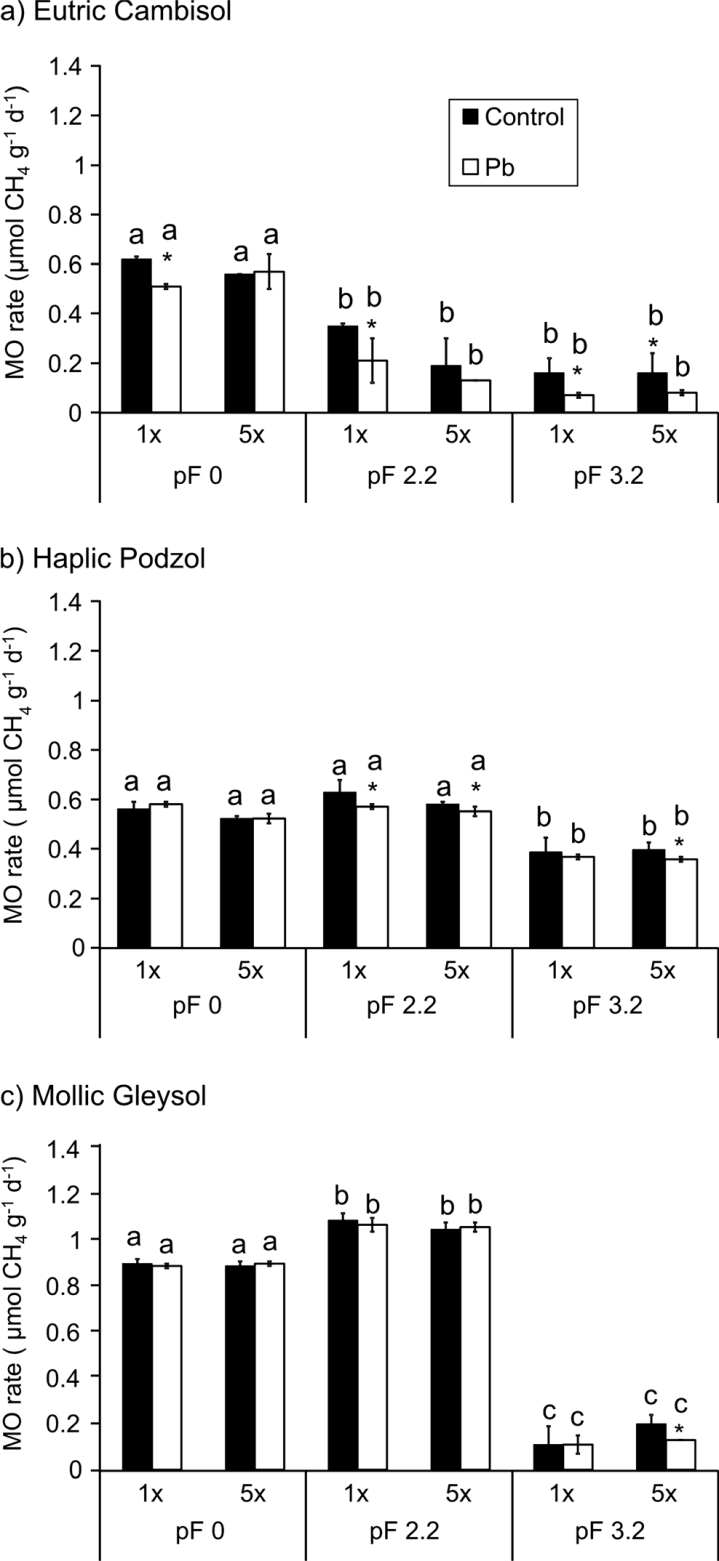
Methane oxidation (MO) rates in the tested soils: **a** Eutric Cambisol, **b** Haplic Podzol, **c** Mollic Gleysol in the control and soils contaminated with Pb at three moisture levels pF = 0, pF = 2.2, and pF = 3.2. Heavy metal was added in two doses: the maximum permitted dose of the metal (Pbx 1) and the fivefold higher dose (Pbx 5). The final concentration of Pb in each soil was 11.6 and 30.8 mg kg^−1^ in Eutric Cambisol, 7.1 and 26.3 mg kg^−1^ in Haplic Podzol, and 12.2 and 31.4 mg kg^−1^ in Mollic Gleysol (asterisks mean a significant difference from the adequate control; the same letter indicates no statistically significant difference among the variants; averages ± SD, *n* = 3; Kruskal–Wallis test; *P* < 0.05)

The average methane oxidation rates (presented in Fig. 2) were calculated based on CH_4_ consumption during the incubation time. This parameter depended on the dose of Pb, moisture, and physicochemical properties of soil. In most samples, the methane oxidation rate was only slightly changed by the addition of the heavy metal, but we observed differences caused by the soil water content.

In Eutric Cambisol, the highest reduction of the average methane oxidation rate in the contaminated soil was noted in the samples at pF = 3.2, in comparison to the control (twofold lower) (Fig. 2a). The higher the moisture, the smaller the differences between the samples were. The greatest values of the methane oxidation rate were obtained at pF = 0, where all methane was completely oxidized (Fig. 1a). The values ranged between 0.62 ± 0.01 and 0.56 μmol CH_4_ g^−1^ d^−1^ in the control and from 0.57 ± 0.07 to 0.51 ± 0.01 μmol CH_4_ g^−1^ d^−1^.

In Haplic Podzol, the highest values of the methane oxidation rate were obtained at pF = 2.2 (Fig. 2b); they ranged between 0.65 ± 0.05 and 0.60 ± 0.01 μmol CH_4_ g^−1^ d^−1^ in the control and from 0.59 ± 0.01 to 0.57 ± 0.02 μmol CH_4_ g^−1^ d^−1^ in the contaminated samples.

In Mollic Gleysol at pF = 0 and pF = 2.2, much higher average methane oxidation rates were obtained than in the other tested soils (Fig. 2c) The highest methane oxidation rate values were also obtained at pF = 2.2, as in the case of Haplic Podzol. The lowest values were obtained at pF = 3.2: 0.11–0.20 μmol CH_4_ g^−1^ d^−1^ in the control and 0.11–0.13 μmol CH_4_ g^−1^ d^−1^ in the contaminated samples, where methane was not oxidized completely.

Moisture influenced oxygen consumption during the incubation of the soil samples. The addition of the heavy metal did not exert an effect on O_2_ depletion, because similar concentrations of this gas were observed in the headspace of the controls and samples contaminated with Pb at the end of incubation. The lowest final O_2_ concentrations, observed in Haplic Podzol, were 13.52% ± 0.67 (pF 0), 13.33% ± 1.06 (pF 2.2), and 13.51% ± 0.53 (pF 3.2) (*v*/*v*). In Eutric Cambisol, with the increasing soil moisture, a lower final O_2_ concentration was noticed, i.e., 13.58 ± 0.32%, 14.73 ± 0.53%, and 15.59 ± 0.4% (*v*/*v*) for pF 0, 2.2, and 3.2, respectively. In Mollic Gleysol, the lowest O_2_ concentration (14.73 ± 0.25% *v*/*v*) was observed at pF 2.2, which resulted from total methane oxidation. At pF 0, the final O_2_ concentration was 15.24 ± 0.11% *v*/*v*, and the highest concentration was observed at the lowest moisture level, which corresponded to pF 3.2 (17.23 ± 0.20% *v*/*v*).

## Discussion

As one of the most toxic environmental pollutants, heavy metals can change the structure and activity of the soil microbial community. Generally, such contamination exerts an inhibitory effect on soil microorganisms (Hassen et al. 1998) by blocking essential functional groups, displacing essential metal ions, or modifying the active conformations of biological molecules (Gadd and Griffiths 1977; Doelman et al. 1994). However, at a relatively low concentration, some metals are essential for microorganisms (e.g., Co, Cu, Zn, Ni), since they provide vital co-factors for metallo-proteins and enzymes (Doelman et al. 1994). In turn, Pb is potentially toxic for microorganisms (Sobolev and Begonia 2008; Markowicz et al. 2010). The response of soil microbial communities to heavy metals depends on the concentration and availability of the pollutants. The behavior of heavy metals in soil depends on many factors, like redox potential (Eh) (Chuan et al. 1996). Moisture changes the oxidation-reduction status controlling the mobilization of heavy metals (Calmano et al. 1993), which affects their concentration in soil solution and bioavailability. It was observed that when the redox potential was decreased (more reducing condition occurs), the solubility of lead was increased (Chuan et al. 1996).

Methane consumption in the tested soils followed Michaelis–Menten kinetics (Walkiewicz et al. 2012), which was confirmed in previous studies (Bull et al. 2000; Gulledge and Schimel 1998; Gulledge et al. 2004; Wang et al. 2011). Based on that experiment, we added 1% of CH_4_, because it was confirmed that the soils did not oxidize the ambient CH_4_ so did not express high affinity activity. The kinetics of CH_4_ consumption may be changed by soil water content as a result of (1) lower O_2_ availability for microorganisms at higher moisture and (2) changing gas concentration because of CH_4_, O_2_ solubility in water (Morris and Schmidt 2013; Stȩpniewski et al. 2005). A consequence of both processes mentioned can be the presence of anaerobic soil microsites in the conditions of higher moisture. Such microsites are a biological source of CH_4_. Consequently, such conditions may be better for low affinity methanotrophs, because higher than ambient concentration of CH_4_ may be available. Simultaneously, higher water content is connected with a lower O_2_ concentration. However, it was confirmed that methanotrophic bacteria may be active under hypoxia (Chistoserdova 2015; Hernandez et al. 2015) even more than under the ambient O_2_ level (Walkiewicz et al. 2016). Wang et al. (2011) confirmed that low affinity methanotrophs prefer low oxygenation which was observed in our experiment with Eutric Cambisol.

The results obtained in our experiment (Figs 1 and 2) show that the effect of the investigated factors on methanotrophy should be discussed in three aspects: (i) the absence of an influence of lead contamination on methanotrophic activity at the beginning of the incubation; (ii) inhibition of this activity after some days in some cases; and (iii) soil moisture (and resulting anaerobic conditions) as an important factor influencing methane oxidation.

To sum up our results, it should be pointed out that the assumed contamination levels did not influence the methanotrophic activity in the first period after lead contamination (Fig. 1.). Practically, in all soils at all water contents, there were no differences between the control and samples with Pb during the first 7–8 days. After this period, inhibition of methane oxidation was observed in Eutric Cambisol and in the driest Mollic Gleysol. However, these inhibitions were not spectacular. In other cases, the influence of Pb contamination was still unobservable.

The absent or relatively small influence of Pb on methanotrophic activity can be explained in three ways (they are not mutually exclusive). Firstly, the initial concentrations of lead in the soils should be pointed out (Table 1.), i.e., before the contamination in the experiment. These concentrations were not high; however, it can be assumed that methane-oxidizing bacteria adapted to the presence of the heavy metal in soil. The second explanation is the natural tolerance of methanotrophs to lead contamination (at least in the range of the investigated concentrations). The third explanation can be the presence of anions (e.g., sulfides) that can reduce the concentration of free lead cations by binding the metal into a sparingly soluble compound. In such a situation, even a small increase in the methane oxidation rate is possible in contaminated soil in comparison to the respective control. There are still no investigations confirming or negating this theory (Mohanty et al. 2014).

The absence or small slowdown of methane oxidation in the presence of lead was shown previously by other reports. Contin et al. (2012) tested the effect of two Pb concentrations on methane oxidation. The results of the research showed that even the addition of 1000 μg Pb g^−1^ had no influence on the process of methanotrophy. What is more, Nies (1999) reported that lead was not as toxic to soil microorganisms as it was considered, and lead-tolerant bacteria were isolated. At this point, our research is novel. We investigated the effect of two stress factors on the soil methanotrophic activity simultaneously: heavy metal (Pb) contamination and diverse moisture levels, which create varied aerobic conditions. The result showed that changes in the concentration of lead in the soil environment did not affect the activity of methanotrophs as much as the soil moisture did. Also Hiltbrunner et al. (2012) found that methanotrophic bacteria were resistant to lead contamination and, as shown by these authors, soil moisture had a much greater influence on the methane oxidation process.

Methane consumption in soil may be affected by different soil properties. Ammonium is common inhibitor of CH_4_ oxidation, mostly in fertilized soils (Gärdenäs et al. 2014). In the tested soils, its initial concentration was at the natural level (Table 1). Despite the presence of such a low NH_4_^+^ concentration, the control soils completely consumed the added CH_4_. Based on that, we suppose that ammonium in the tested soils should not affect CH_4_ consumption. Additionally, the tested soils were characterized by low salinity. Another reason of the differences in the process of methane oxidation in the soils used may be caused by the differences in the organic carbon content of each soil, which may have an influence on the effect of Pb contamination on the process. Many investigations have shown that the lead uptake in soil is strongly correlated with soil pH and organic matter (Gao et al. 1997). In our experiment, three soils with similar pH were used. That is why, it is supposed that the main reason of the discrepancy in the described process is caused by the formation of Pb complexes with organic matter (Jensen et al. 2006). According to literature, the activity of Pb in soil was lower when soluble complexes with fulvic acids were formed (Ge et al. 2005). What is more, another research has demonstrated that humic acid has higher capacity to bind Pb ions in complexes, which results in lower toxicity in soil (Jordan et al. 1997). As suggested by Reicosky (2005), the higher the organic matter content, the higher the ability of soil to hold water. This is possible because of formation of more micro- and macropores, which create favorable living conditions for soil microorganisms. Similarly, the phenomenon of progressive inhibition of methanotrophs during the incubation time in tested soils was observed by other authors. For instance, Khan et al. (2010) showed that the greatest inhibitory effect of heavy metals on microorganisms was observed after 2 weeks of incubation.

The amount of water in the soil (or more precisely—the number and size of soil pores filled with water) influences two crucial factors that are important for the activity of soil microorganisms, including methanotrophs (Singh and Kashyap, 2007). Water is the natural environment for bacterial life. They are active in the space between soil particles filled by water or in water films on soil particles or aggregates (Gebhardt et al. 2009; Fest et al. 2016). On the other hand, the soil moisture content determines gas diffusion, which is the basic mechanism of gas exchange (Stȩpniewski et al. 2005). At low soil moisture, where oxic conditions are present, methanotrophic bacteria consume CH_4_ (Curry 2007). When the moisture rises, the oxic conditions are limited, and this activates methanogenic Archaea to produce methane. This results in the release of CH_4_ to the atmosphere without oxidation. The results presented in our work confirm this general statement but show that, in some cases, the higher water content is not a limiting factor for methanotrophs. Due to the highest water content, anaerobic methane oxidation may occur in soil microsites. Nitrate (NO_3_^−^) may be an alternative electron acceptor in non-oxygen conditions (Gardiner and James 2012).

The most “classical” results were obtained for Mollic Gleysol. It can be seen from Figs 1 and 2 that the highest efficiency was obtained for pF 2.2. Lower methanotrophic activity was noted at the highest moisture levels (pF 0) and dry soil (pF 3.2) was characterized by the significantly lowest activity. A slightly less spectacular but similar relationship was seen for Haplic Podzol. This is consistent with the theory presented by Nosalewicz et al. (2011), who stated that the optimum soil moisture for methanotrophs is close to half the value of soil water capacity. However, the fastest oxidation of methane in Eutric Cambisol occurred at the highest moisture level (pF 0). This may be caused by the presence of methanotrophs in this type of soil for which the proposed moisture, and thus the specific oxygen requirements, are optimal for activity. It can be assumed that such bacterial species are not present in the other two types of soils or their number is not sufficient to carry out a process with a capacity such as in Eutric Cambisol.

Boeckx and Van Cleemput (2000) found that the most efficient soil moisture for methanotrophy is between 15.6 and 18.8% *v*/*v*. Very similar values were given by Whalen et al. (1990), who tested soil samples consisting of sand mixed with two types of clay, brown and gray, collected from a park. Based on the tests, it was found that the optimal soil water content for the highest methanotrophic activity was ca. 11%. The proposed moisture is the optimal amount of water required for enhancement of methanotrophic activity and for gas diffusion on the surface of the bacterial cell. Moisture of pF 2.2 used in our tests (13%) is in the range of values given by these authors. Different values were given by Castro et al. (1995), who found that the optimum water content for methanotrophy in mineral soil collected from a pine plantation was from 20 to 60% *v*/*v*, where the consumption of methane was up to 0.25 mg CH_4_-C m^−2^ h^−1^. We have no detailed information about the soil used in the experiment described by Castro, but taking into account the results obtained for our Eutric Cambisol in which higher moisture was more appropriate for methanotrophy, we can assume that it is a similar case.

Depending on the soil moisture and Pb dose, different lengths of the lag phase were observed (7–10 days in Eutric Cambisol and 2–3 days in Mollic Gleysol and Haplic Podzol). We suppose that this can be connected with the presence of different species of methanotrophs, which need different lengths of time for multiplication and adoption to current conditions. This is in agreement with the results reported by Syamsul Arif et al. (1996), who confirmed the length of the lag phase of several days. In cultivated soils, the lag phase can last even 2–3 weeks because of NPK fertilization (Hütsch 2001). In tested soils, the initial content of NPK (Table 1) was the highest for Mollic Gleysol. The initial nitrogen concentration was at a natural level (0.08–0.17%); therefore, it should not significantly influence methane oxidation although N is a strong regulator of methanotrophic bacterial activity in arable soils (Bodelier and Laanbroek 2004).

### Conclusions

We investigated the methane oxidation process in selected mineral soils under different water contents and Pb contamination. The water content was a stronger factor regulating methanotrophy. Methanotrophic bacteria showed tolerance to Pb at the beginning of incubation. A stronger inhibitory effect of Pb was observed in the second week of incubation. The most favorable conditions for methane-oxidizing bacteria in Eutric Cambisol were observed at pF 0. In Haplic Podzol and Mollic Gleysol, reduction of the moisture content from pF 0 to pF 2.2 resulted in a slight increase in the methane oxidation rate. The fastest CH_4_ oxidation was observed in Mollic Gleysol, which was influenced by the highest content of organic matter in comparison to the other two tested soils.
